# Social Capital and Cultural Health Capital in Primary Care: The Case of Group Medical Visits

**DOI:** 10.1111/1467-9566.13868

**Published:** 2024-12-16

**Authors:** Ariana Thompson‐Lastad, Jessica M. Harrison, Janet K. Shim

**Affiliations:** ^1^ Department of Family and Community Medicine Osher Center for Integrative Health University of California San Francisco California USA; ^2^ Osher Center for Integrative Health University of California San Francisco California USA; ^3^ Department of Social and Behavioral Sciences University of California San Francisco California USA

## Abstract

This article focuses on an empirical setting that upends the clinician–patient dyadic norm: group medical visits (GMVs), in which multiple patients gather in the same space for medical care, health education and peer support. Our grounded theory analysis draws on participant observation and interviews (*N* = 53) with patients and staff of GMVs at four safety‐net healthcare organisations in the United States. We delineate (1) how group medical visits provide health‐focused social networks that facilitate the mobilisation of social capital, (2) how the organisationally embedded relationships that comprise group visits are made possible through extended time that is part of the GMV field and (3) how clinicians have opportunities rarely found in other settings to learn from patients, using knowledge accrued from GMV networks to advance their own skills, thereby converting social capital into provider cultural health capital. GMVs provide a rich empirical site for understanding the ways in which organisational arrangements can shape opportunities for patients and clinicians to cultivate and mobilise social capital and cultural health capital, and in doing so, materially shift experiences of receiving and providing healthcare.

## 1 Introduction

Much healthcare takes place within the interaction between a patient and clinician dyad. In contrast, group medical visits (GMVs) bring multiple patients together with one or more clinicians for simultaneous medical care and health education. Moreover, GMVs are intentionally organised to facilitate peer social support among patients. Given their orientations to social interaction in the medical context, we argue that GMVs have distinct theoretical and empirical value for understanding sociological processes, including the accumulation and mobilisation of different forms of capital in healthcare and the influence of institutionalised relationships and social position on how capital is acquired and used.

Group medical visits are increasingly widespread in primary and speciality care (Ickovics et al. 2017; Parikh et al. 2019) and have been implemented in a wide range of healthcare settings across the world. In the United States, GMVs are often offered in community health centres, which are key institutions in the healthcare safety net, providing primary care for poor and working‐class people (Thompson‐Lastad, Gardiner and Chao 2019). Where typical US primary care visits are critiqued for being 10–15 min long (Fiscella and Epstein 2008; Satterwhite 2019), GMVs last between 1 and 3 h per session. GMVs represented in our study varied in duration, frequency of meetings and stability of membership. Some met weekly for six or eight sessions, while others had gone on for years. Some were gender‐specific or focused on a particular health condition; others had broad criteria for participation. However, many key elements were consistent: these GMVs were for adults with chronic conditions, commonly including chronic pain, diabetes and high blood pressure; all included billable medical care from a clinician and also health education, peer support and complementary and integrative therapies (e.g., acupuncture and yoga). Participants' health concerns shaped the specific activities of each group, as did clinician and administrator decisions.

In the emerging medical literature on group visits, there is growing evidence of their health and social impacts. GMVs provide institutionalised spaces where patients can develop social support *within* and *during* the provision of care in health‐related community with peers (Thompson‐Lastad 2018; Geller 2019). Studies have generally shown that GMVs result in health outcomes similar to or better than individual care (e.g., Papoutsi et al. 2017; Chao et al. 2019; Gardiner et al. 2019; Vaughan et al. 2020), while offering additional, intentional benefits from clinic‐based peer interactions (Kennedy et al. 2009; Bruns et al. 2019; Geller 2019). For example, research on group prenatal care has found promising results in Malawi, Nepal, Sweden and Canada, among other countries (Patil et al. 2017; Harsha Bangura et al. 2020; Sadiku et al. 2023). In the US, a recent study of group prenatal care at two organisations in the state of Tennessee found more positive mental health outcomes among patients with high levels of group participation (Kettrey and Steinka‐Fry 2021). Advocates of GMVs in the US have promoted them as a strategy to narrow health disparities and increase patient‐centred care (Burnett and Truesdale 2019; Geller 2019).

In this paper, we shift focus from group visit outcomes to processes and mechanisms, and offer a fine‐grained analysis of the kinds of interactions afforded by GMVs. Using observational and interview data from a mixed methods study of GMVs in US community health centres, we approach GMVs as emergent social networks focused on health. We examine how the group setting illuminates the mobilisation and transmission of different forms of capital. We argue that the presence of peers in healthcare encounters can affect the cultivation and mobilisation of social capital (Lin 2005; Bourdieu 1983) as well as cultural health capital (Shim 2010). As a dense, organisationally specific network where people meet regularly, GMVs can cultivate health‐related social support and information exchange in ways that are rare in healthcare and distinct from dyadic patient–clinician interactions (Lavoie et al. 2013; Housden et al. 2017; Thompson‐Lastad 2018).

## 2 Background

### 2.1 Field and Habitus

To understand how social capital and cultural health capital operate in GMVs, it is essential to understand Bourdieu's concepts of field and habitus. Capital exists within what Bourdieu (1991) calls a ‘field of action’. Each field has its own rules and norms, and exists in particular times and spaces. These norms and rules are shaped in turn by broader social structures and power dynamics outside of the specific field. Social stratification in a society shapes what kinds of stratification exist in a given field, and the field exists outside of individuals and their practices and preferences. As Chang, Dubbin, and Shim (2016, 93) write, fields ‘reflect the social structures and power relations of the people in the field. A person's experience in and capacity to navigate a field depends on her status and position in these structures and relations’. The rules of the field in turn shape what forms of capital are valuable, and how capital is distributed (Bourdieu and Wacquant 1992). Those people with more of the kinds of capital valued in a given field are those who hold more power.

Social scientists have identified healthcare and biomedicine as distinct fields, which have ‘permeable borders’ (Collyer 2018). They are shaped by broader economic and political fields, which shape biomedicine in varied ways depending on place and time (Doblytė 2019). In the battle for social status that is inherent to a field, ‘those who are able to determine what resources hold value in the first place possess the power to set the rules of the game’ (Dubbin, Chang, and Shim. 2013, 115). Within biomedicine, physicians generally hold highest status and set the rules for interaction. These norms and rules of healthcare differentiate between patients and clinicians by affording great significance to professional status, expertise and credentialed knowledge held by physicians and other clinicians.

Bourdieu uses the term habitus to describe the deeply embedded and habituated ways that people think, act and look at the world. He defines habitus as ‘systems of durable, transposable, dispositions … principles which generate and organise practices and representations’ (1990, 53). For individuals, habitus shapes what is perceived and how, and the ways people go about accomplishing tasks. Importantly, these dispositions arise out of ‘[t]he conditions associated with a particular class of conditions of existence’ (1990, 53), and so people in a shared social context may have a similar habitus, with variations depending on their individual biography (Bourdieu and Nice 1977). Habitus is produced by social context, embodied by individuals, and though durable, can shift over time. In a specific field, then, habitus shapes people's experiences, their understandings of the prevailing rules and norms of the game, and their ability to navigate that field. Spending time in a field can allow someone to learn the rules of the game and, over time, lead to shifts in habitus.

Habitus shapes how both patients and clinicians arrive in a healthcare setting, and what they expect from the other people they interact with. For patients, habitus shapes what kind of care people seek for health concerns, and how they engage with healthcare (Doblytė 2019). Clinicians assess patient habitus in determining how to interact with someone and what kind of care to provide, though they may be unaware that they are doing so (Chang, Dubbin, and Shim 2016). Importantly for our article, biomedical clinicians can also be understood to possess a general habitus; Bourdieu describes a shared habitus—a ‘community of dispositions’ (1977, 79)—that can come not only from shared personal experiences but also from professional training. Elements of clinician habitus include personal and social characteristics, individual histories as well as clinical training and socialisation that together shape how they provide care (Chang, Dubbin, and Shim 2016; Berndt and Bell 2020).

### 2.2 Cultural Health Capital

Shim (2010) developed the concept of cultural health capital (CHC) to account for how macro‐level social hierarchies shape and express themselves in clinical encounters, and how micro‐level healthcare interactions accrete to (re)produce unequal care, in patterned but not wholly determinative ways. CHC is rooted in Bourdieu's notions of field, habitus, and of course, cultural capital. Bourdieu (1983, 1990) defined cultural capital as socially transmitted and consecrated resources, practices and goods (e.g., educational credentials, knowledge of cultural domains and styles of speaking) that confer advantages for social status and distinction within a field. Shim (2010) applies this concept to the field of healthcare, where CHC is a specialised form of cultural capital that can be mobilised in clinical encounters to generate more attentive and mutually satisfying patient–clinician interactions.

Elements of CHC that Shim identified as generally important in the current US context include health literacy, a proactive attitude towards disease management, the ability to communicate health‐related information efficiently and the organisational savvy to navigate complex institutions like healthcare. Although we may think of contemporary biomedical care broadly as a field, its organisational complexity means that it comprises many related but not identical fields. Thus, specific aspects of CHC may be more or less helpful in different healthcare settings, with different clinicians at different times (Shim 2010; Madden 2018; Rubin et al. 2018); what constitutes CHC in any given healthcare interaction is organisationally and situationally specific. Its value is determined in large part by gatekeepers: though patient–clinician interaction is always bidirectional, clinicians determine what skills and interaction styles matter most, and set the rules of clinical interaction (Dubbin, Chang, and Shim 2013). For example, Shim (2010, 10) points out that ‘certain dispositions might be an asset in the outpatient clinic but not in the emergency room, or some skills may be advantageous in healthcare institutions serving middle‐class, privately insured populations, but not so in resource‐poor county hospitals’. These institutionalised norms of the kinds of CHC that clinicians expect and reward, therefore, help to distinguish the particularities of the specific healthcare fields in which patients and clinicians encounter one another, explain how interactional processes unfold, cascade and accumulate into systemic inequities in care.

Of particular relevance for this paper is that clinicians can also be understood to acquire, possess, and leverage CHC. When understood as resources and skills that ease healthcare interactions, CHC can be accumulated and deployed by clinicians as well as patients (Chang, Dubbin, and Shim 2016). Existing research suggests that some elements of CHC are common to both clinicians and patients, for example, ‘good communication skills … and the ability to adapt one's interactional style’ (Chang, Dubbin, and Shim 2016, 94).

In line with Bourdieu's understanding of habitus, components of CHC comprise a coherent collection of skills or a ‘tool kit’ that may be purposefully and strategically used, but more often are tacit, habituated ways of thinking and acting in healthcare interactions. CHC, like other forms of capital, requires the investment of time and effort, as it is acquired through the repeated enactment of health‐related practices and use of cultural resources. It is through these mechanisms that CHC is tied to social stratification and inequality in healthcare. Habitus originates out of status‐specific conditions of life (Bourdieu 1990). Because cultural capital (and CHC) require both time and the opportunity to cultivate and accrue culturally legitimated (and health‐related) competence, dispositions, and behaviours, CHC redounds and compounds itself (Shim 2010). In this sense, the time afforded in patient–clinician interactions are a linchpin to either reproducing or countering the stratified nature of CHC and its consequences for healthcare inequalities. Writing about chronic pain care, Rubin et al. (2018) describe how extended time in a clinical setting allows healthcare workers greater opportunities to cultivate patients' CHC. Given the time‐ and energy‐intensive nature of cultivating CHC, healthcare settings that are subject to constrained time for patient–clinician interactions ‘are likely to curb providers' ability to work with patients to maximise the CHC available in the clinical encounter’ (Shim 2010, 6). Conversely, care settings such as group visits—where much longer and more flexible clinical time is in fact organisationally mandated and institutionalised—offer the possibility for those providing and seeking care to surface, realise and use available cultural knowledge.

### 2.3 Social Capital

Bourdieu's definition of social capital focuses on actual and potential resources that inhere in social networks (Bourdieu 1983). Lin (2005, 51) defines social capital as ‘resources embedded in one's social networks, resources that can be accessed or mobilised through ties in the networks’ to benefit individuals, while Carpiano (2006, 166) describes it as ‘collective resources of groups that can be drawn upon by individual group members for procuring benefits and services’. These definitions share a focus on the social networks one is connected to and the resources that network members possess, that can then be mobilised by individuals.

Social capital has been studied in relation to multiple health outcomes (Ehsan et al. 2019), and to a lesser extent within healthcare settings (Pitkin Derose and Varda 2009). Elaborating on the specific aspects of social capital that provide health advantages, Carpiano (2006) proposes that social support and social leverage are key forms of social capital that help individuals access beneficial information. Perry and Pescosolido (2015) identify the exchange of health information as one important way in which networks provide benefits beyond those of each individual relationship. Reich (2020) highlights multiple ways that social networks relate to health: by providing social support, support with self‐improvement efforts, and informational support. The importance of knowledge and information for navigating medical issues also underscores the consequences of the unequal distribution of social capital, given that social status stratifies the information and power possessed by social networks (Lin 1999, 2005; Small 2010). For example, Hernandez and Calarco (2021) find that individuals' social status shapes the kinds of information they receive from social networks and clinicians.

Bourdieu's concept of social capital focuses specifically on ‘more or less institutionalised relationships of mutual acquaintance and recognition—in other words, to membership in a group’ (1983, 248), that provides the context for ‘a continuous series of exchanges in which recognition is endlessly affirmed and reaffirmed’ (1983, 250). Small and Gose (2020) have examined how such institutionalised relationships are forged in organisational settings, such as childcare and community centres. Their research discusses how organisations can ‘help low‐income people to form new social ties’ (2020, 91), which lead to increased social capital. They suggest that the success of such efforts ‘depend[s] on the extent to which institutional norms render interaction frequent, long‐lasting, focused on others, or centred on joint tasks’ (2020, 92). These institutionally embedded interactions can lead to the formation of social ties that are strong but also domain‐specific, what terms ‘compartmental intimates’ who share ‘a relation characterised by openness, trust, and the revelation of privacy, but only within confined domains’. Analysis of the cultivation of social capital through networks forged within organisations is especially relevant to group medical visits.

Bourdieu (1983) noted the connections between social and cultural capital, stating they can be mobilised together to access resources, and can each be converted into economic capital. More recently, scholars have elaborated on Bourdieu's assertions of the convertibility among different forms of capital, to explore the interactive effects of social and cultural capital. Lizardo (2006) reveals how possessing a diversity of cultural knowledge can be converted into distinctive kinds of social connections (e.g., strong and weak ties). McConnell (2017), following Lizardo (2006; also Bourdieu 1983), demonstrates a capital conversion model in which the level of social support that individuals mobilise from their networks depends on the specific cultural resources those network members hold. McConnell (2017, 516) found that people with trust in physicians *and* past mental healthcare experience were more likely to provide emotional and instrumental support to people with newly diagnosed mental health concerns, who ‘have the chance to convert not only their own capital but also the cultural capital of their network members into supportive social resources’. The interactive effects of cultural and social capital also pertain to healthcare professionals. In a study of community health centres, for example, Madden (2018) discusses how clinic staff used flexible and localised forms of CHC, such as their knowledge of structural barriers to healthcare access, as well as their own social capital in professional networks, to help patients access care from specialists. Thus, in situationally specific ways, the cultural capital of those one is connected to can be converted into social capital, and social capital can influence and shape cultural resources (see also Doblytė 2019).

### 2.4 Field, Habitus and Capital in Group Medical Visits

Group medical visits are situated within the field of biomedicine, with overlapping interactional rules and many of the same norms (e.g., use of electronic health records). What it means to be a ‘good GMV clinician’ or a ‘good GMV patient’ has a great deal of overlap with other biomedical settings. However, GMVs have distinct rules of the game, including extended time and the presence of peers participating in one another's care (Thompson‐Lastad 2018). Patients are invited to participate, for example, by checking their own blood pressure in many GMV settings. GMV patients share knowledge, experiences and support, while often being less deferential to credentialed clinicians.

For clinicians who are used to practicing standard biomedical care, the norms in a GMV setting are distinct (Teate, Leap, and Homer 2012; Thompson‐Lastad 2018). Relationships between patients and clinicians are typically more egalitarian, and clinicians' communication usually includes a facilitative leadership style and more self‐disclosure than is common in individual care. When clinicians train to practice in GMVs, some describe departing from the established doxa of biomedicine, learning to let patients talk with their peers rather than immediately providing a clinical response to questions. The explicit goals of GMVs vary and make visible the ‘porous borders’ of a field (Collyer 2018). For example, some GMVs focus on providing standard prenatal or diabetes care in an efficient fashion, while others prioritise expanding access to integrative therapies such as acupuncture or yoga that are not widely available in biomedical settings.

Group visits provide a unique window into the circulation and conversion of social and cultural capital because of the preponderance of patient‐to‐patient interactions that would be unlikely or impossible to occur in individual visits. In what follows, we argue first that GMVs provide health‐focused social networks that facilitate the mobilisation of social capital through patients' mutual sharing of informational resources and the value accorded to patients' experiential knowledge. Second, we show how the institutionalised, organisationally embedded relationships that comprise GMVs, and the repeated encounters and extended time of GMVs, make it possible for all parties to cultivate cultural health capital and become ‘compartmental intimates’. Finally, we find that clinicians have opportunities rarely available in other settings to learn from patients, and use knowledge accrued from GMV networks, thereby converting social capital into clinician CHC.

## 3 Data and Methods

This article is part of a larger, mixed‐methods study that examines how group medical visits and integrative healthcare were combined and implemented in four community health centres in the San Francisco and Boston areas. Study clinics were chosen for their robust GMV programs; all had offered group visits for at least 10 years. Data were collected during 2015–2016 and included ethnographic observations of 21 group visits across eight sites, as well as interviews with group visit patients (*n* = 25; see Table 1) and clinic staff (*n* = 28; see Table 2). Group medical visits typically are facilitated by multiple staff members, including a licenced clinician (e.g., physician or nurse practitioner) and one or more support staff (e.g., health educator or medical assistant).^1^ Fieldwork was conducted by the first author (ATL) in English and Spanish. With clinicians involved in coordinating GMVs at each organisation, ATL identified GMV staff as potential participants. All relevant staff were invited via email or in person to be observed providing care in a GMV, then participate in a semi‐structured interview. Patients and staff provided verbal consent at the time of observation; observations were recorded in detailed field notes.

**TABLE 1:** Demographics of group visit patient sample (*N* = 25).

Characteristic	*n* (%)
Age, mean years (SD)	58 (12)
Sex, *n* (%)	
Male	7 (28)
Female	18 (72)
Race and ethnicity, *n* (%)	
Black/African American	15 (60)
Hispanic/Latina/o	3 (12)
White	5 (20)
Other racial identity	2 (8)
Education, *n* (%)	
Less than high school	2 (8)
High school	7 (28)
Some college	9 (36)
Associate or bachelor's degree	5 (25)
Self‐reported chronic conditions, *n* (%)	
Diabetes	7 (28)
Chronic pain	18 (72)
Mental health condition (most common depression, PTSD)	10 (40)
Hypertension	7 (28)
3 or more chronic conditions (including conditions listed above)	12 (40)
Length of participation in group visits, *n* (%)	
< 6 months	13 (52)
1–2 years	8 (32)
> 2 years	4 (16)

**TABLE 2:** Demographics of group visit staff sample (*N* = 28).

Characteristic	*n* (%)
Age, mean years (SD)	43 (SD 12)
Sex, *n* (%)	
Male	6 (22)
Female	22 (78)
Race/ethnicity, *n* (%)	
Asian	3 (11)
Black/African American/African	2 (7)
Hispanic/Latina/o	4 (14)
White	15 (54)
Multiracial or other	4 (14)
Primary role, *n* (%)	
Physician	13 (46)
Manager or programme coordinator	4 (14)
Health educator/group visit coordinator	4 (14)
Other licenced clinicians (nurse‐practitioners, psychologists)	2 (7)
Other support staff (medical assistant, substance abuse counsellor, promotora, AmeriCorps member)	5 (18)
Years of experience in group visits, *n* (%)	
< 1 year	2 (7)
1–5 Years	14 (50)
6–10 years	6 (21)
> 10 years	6 (21)

ATL conducted 28 staff interviews. The content was iteratively adjusted to explore themes in ethnographic observation and patient interviews. While observing group visits, ATL typically invited all patients to participate in individual interviews. ATL interviewed 25 patients by phone or in person. Interviews generally lasted 1 h and focused on patients' experiences in GMVs, including relationships with staff and other patients. All interviewees provided written consent and received a $25 gift card. Interviews were audio‐recorded. Demographic data were collected through a brief questionnaire.

Interview transcripts and field notes were professionally transcribed, then analysed using grounded theory methods, including coding and memoing (Clarke 2005; Charmaz 2014). All data were coded using qualitative data management software Dedoose in the original language of data collection. During open coding, ATL read all transcripts and field notes multiple times, then inductively developed a list of initial codes. The code list was adjusted as additional materials were coded, then refined through focused coding in areas including patient–clinician and peer relationships. Ongoing memo writing and discussion with co‐authors JMH and JKS addressed preconceptions about how social and cultural capital might be relevant to this project. This process was especially important as ATL had been a GMV co‐facilitator at one of the research sites prior to data collection. Selected categories related to staff experience of GMVs, knowledge exchange, peer relationships and characteristics of ‘good’ GMV clinicians were used for this article. Study procedures were conducted with the approval of the relevant institutions' IRBs.

## 4 Results

### 4.1 Group Medical Visits as Networks for Social Capital Sharing

Group medical visits, by their very structure and format, cultivate the formation of health‐focused social networks. Group visits are organised this way intentionally: they are longitudinal programmes that provide extended time within and across sessions, and encourage communication among people who might not otherwise meet. They typically include a pair of clinic staff and between 6 and 15 patients. This structure allows patients to share experiences, and staff to recognise and facilitate these opportunities. Staff and patients alike recognise the value of patients' accumulated knowledge about health conditions and community resources, their skills and lived experience in managing medical issues and the emotional value of providing support. These resources constitute the social capital of the group, including ongoing relationships with healthcare workers who themselves generally have high social capital.

When healthcare interactions include multiple patients, each has the potential to share and receive health‐related knowledge with and from other participants, benefitting from other patients' habitus and past healthcare experiences. This multidirectional interaction is not possible in a traditional visit. GMV staff see knowledge‐sharing among patients as part of their facilitation role, making space for patients to support one another, acquiring and mobilising social capital. In one group, the clinician, Adisa, facilitated brief check‐ins with each patient. A patient named Loretta shared that she was getting ready for surgery; another patient reminded the group that Loretta would be getting her cataracts removed. After Adisa briefly explained what cataracts are, another patient, Margaret, said her cataract surgery was wonderful—now she could see ‘all those beautiful houses on top of the hill!’ Adisa asked Margaret to describe the surgery, asking, ‘Do they give you any calming medicine?’ ‘Oh yes’, Margaret answered. Adisa reminded Loretta to make sure they do surgery on the correct eye, and ‘ask the doctor what you can and can't do’ after the surgery. Margaret urged her to use eye drops regularly after surgery.

We can view this kind of knowledge‐sharing among patients and staff as a relatively straightforward example of the mobilisation of social capital. Loretta benefits from the group's social capital because Margaret shares her knowledge of and experience with cataract surgery. Loretta can access others' social capital because of her membership in the GMV social network. In this case, Loretta can leverage her relationships with group members to obtain new information about and interactional pointers for her cataract surgery, and is likely to deploy this capital for a better outcome than might have happened otherwise. Loretta shared that she felt more prepared from her surgery after the conversation with Adisa and Margaret and broader group support. We also underscore how this social capital exchange is facilitated by the GMV structure, which provides access to new relationships and time for direct communication between patients with others listening. It is another patient who reminds the group of the specific surgery that Loretta is having; another patient, Margaret, volunteers how significantly cataract removal improved her vision; she is then invited to elaborate on her experience of the surgery. While it is the clinician who defines what cataracts are and prompts Margaret to share further, GMV facilitators understand that part of their role is to actively promote the formation of relationships among patients. This sanctioning of social capital formation and leveraging of those relationships for health‐related benefits allows patients to articulate questions and obtain answers from each other in relatively unconstrained ways.

In a men's health group co‐facilitated by a clinician, Gabriel, and a support staff member, patients helped each other check their weight and blood pressure, then sat in a circle of chairs to ‘check in’. Gabriel opened, saying, ‘we're all here to work on our health’. Tony, a patient, mentioned plantar fasciitis as a bothersome health concern, and said he was receiving free acupuncture to treat it. Gabriel asked if it was helpful, and Tony said yes. As Tony shared details of the acupuncture programme, Gabriel wrote them on a whiteboard. The next patient to speak explained that he also was receiving free acupuncture, but it was not helping his pain. After a third patient said his plantar fasciitis was resolved with a cortisone shot, Gabriel turned to Tony and offered him a podiatry appointment, which the co‐facilitator would schedule at the end of the session.

In this series of exchanges, Tony shared and received knowledge with other patients after the GMV clinician (Gabriel) provided an opening and support. Tony shared a treatment that he found beneficial, and the clinician made sure the details of how to access it were available to other patients in writing. Though Gabriel mentioned he had been aware of this resource, he had not recommended it to the group. Because Tony brought it up, patients and staff received information about when and how to access a potentially useful treatment. One patient's mention of another treatment that had been helpful to him (a cortisone shot) seemed to encourage Gabriel to offer Tony the same kind of care. In scheduling the appointment, Tony mobilised social capital, using information shared by a group member and acted upon by staff to access a new treatment. None of this could have taken place in an individual visit. The structure of the GMV field, which creates a longitudinal and patient‐centred network, allowed for social capital mobilisation.

Patients described how social capital was transportable, where resources accumulated in one GMV could be used elsewhere. Zoya had participated in a Spanish‐language diabetes GMV years before, then recently began attending another. In an interview, she explained that in the prior group she had learnt about therapy for mental health needs, as well as community resources to address economic stress:

Both the doctor and the patients who participate … we learn things, or we can help other people with things they might not know …. Being in the group was how I was able to realize how to improve my health, not just physically but emotionally. When I started going to therapy, that was where I learnt breathing techniques, and then with the … group visits, I've been seeing when I can use those techniques … Every group … I learn something that helps.

Zoya's initial GMV experience allowed her to leverage social capital and access therapy. In turn, she shared techniques learnt in therapy with fellow patients in a different GMV years later, where I observed her recommending therapy and breathing practices to other patients who were struggling with mental health. Zoya leveraged the social capital of her previous GMV to gain the benefit of therapy for her mental health, then carried it to others in a new group. Social capital can provide spillover benefits from a group interaction at one point in time to another health‐focused social network at another point. We identify these as key and distinctive effects of GMVs.

### 4.2 Building Trust in Group Medical Visits Through Extended Time

One of the ways in which the GMV field differs most from individual care is the extended time and frequent encounters—typically weekly or monthly—that allow for among patients and staff. Extended time in GMVs makes it possible for all parties to cultivate CHC that they can use in other healthcare interactions. The structure of GMVs meant that teaching patients—about a safe blood pressure level, for example, or the benefits of medications for chronic conditions—is done with both adequate time and support from other patients. The benefits of extended time were mentioned in nearly all interviews conducted as part of this study.

Clinicians viewed the extended time of GMVs as allowing them to provide higher‐quality care than in individual visits, mobilising their CHC in ways that align with existing literature (Rubin et al. 2018). Extended time allowed clinicians, staff and patients to build trusting, longitudinal relationships. Rohan facilitated groups for pregnant women and adults with chronic conditions in both Spanish and English. He described time as a key element of developing trusting relationships:

Time is huge because it's building trust. It's continuity. Four 15‐minute visits over the course of a year [in individual care], where I'm rushed and we're talking about all sorts of things is very different than six two‐hour sessions [in group]. Or for a pregnant woman, that bond and connection they feel with me means that, now I can be the doctor for their family … for their kid, and also for their husband, who hasn't seen a doctor in 10 years.

Rohan went on to acknowledge that time alone did not explain the positive effects of GMVs, and spoke about benefits of co‐facilitation, in his case with racially and linguistically concordant community health workers. He called these colleagues ‘the trust link. To call [the patients] and remind them, to follow up on their goals, to show them that the system cares about you’. This too demonstrates extended time as a key element of the GMV field: staff time and flexibility to be available to patients by phone between group sessions, a common element of the GMV co‐facilitator role.

Rohan's experience was that GMVs allowed for relationship‐building in ways that individual primary care in a busy safety‐net clinic could not. This was a nearly universal theme in interviews with patients and GMV staff. Whereas a chronically ill individual patient in Rohan's clinic might spend 60 min with their primary care provider over the course of a year, a patient attending a GMV would spend 12 h or more with a clinician, another healthcare worker, and a group of peers. As part of the distinct GMV field, patients can observe how the clinician interacts with other patients. This can lead to substantial change in patient–clinician interactions and may promote new dispositions over time. Rohan pointed to how trust—once won with a patient—can multiply: there can be spillover effects onto family members trusting clinicians. Additional interviews indicated that perhaps these mended relationships with GMV staff could lead patients to transfer that trust to other healthcare interactions, while also developing self‐advocacy skills through group participation.

This sense of expansive and elastic time is one of the key norms of GMVs. In many cases, being able to collectively share and problem‐solve increased patients' capacity to follow medical recommendations that they understood to be legitimate but experienced as unworkable. For example, Zoya had several chronic conditions that required medication, but before joining the group had felt ‘hopeless, disheartened’ because of problems with medication side effects, compounded by the unavailability of consistent primary care appointments to address those concerns. Zoya had a history of cancer that had been diagnosed at a late stage due to what she described as physicians' negligence, and more recently had been told by an unknown physician that her medication side effects were caused by her weight. When a GMV co‐facilitator called Zoya and invited her to a group, she began attending. By the time of our interview, Zoya had attended several sessions where she said, ‘I feel safer, more cared for’. Participating in the group helped her feel comfortable disclosing that she had discontinued all medications because of unpleasant side effects. Zoya recounted the clinician's empathic response: ‘First, he said that he appreciated my trust in telling him [that I wasn't taking any medication]. He said, “I see that you're aware that you're causing yourself harm … and now we're going to start over”.’ Zoya felt protected from potential clinician judgement by the presence of other patients with similar struggles, and the GMV clinician had adequate time to listen to and understand her concerns. After this interaction, the clinician prescribed medications that did not cause side effects and wrote prior authorisation letters to the insurance company to assure that Zoya had consistent access to these medications. Zoya was relieved and began taking the new medications regularly. Zoya changed her daily health‐related behaviours because the context for them changed, thanks to GMV participation. Her story exemplifies how patients may cultivate cultural health capital, resulting in greater power in relationship with clinicians in GMVs.

Many patients viewed GMVs as providing adequate time to meet social *and* medical needs, in a more relaxed setting, as compared to individual care within the same building. In the field of individual care, clinicians had viewed Zoya as non‐adherent to recommendations. In the GMV, extended time and the patient network provided Zoya with space and support to share honestly about her medication concerns, as well as opportunities to develop new ways to interact with clinicians, increasing her CHC. This unfolding set of interactions display the prevailing norms of the GMV—that patients can directly support one another without worrying about the opinions of clinicians, that clinicians are there to support and not be directive or judgemental, and that there is space and time to air and get to the bottom of health concerns. Zoya's needs for care were met, and her adherence to treatment increased while she supported fellow group members with referrals to community resources and coping skills for mental health symptoms. Many clinicians and patients shared that patients felt more comfortable in GMVs disclosing things that did not align with medical recommendations, while learning skills and information that led them to adhere to recommendations. All of this suggests how time spent in the field of GMVs helps to cultivate particular dispositions (habitus) towards health and specific kinds of CHC.

Emotional support was an important element of social capital facilitated by extended time. Hannah was one of many patients who likened their GMV co‐participants to a family. She said, ‘Everybody opens up and says what's going on with their life … It helps you to put your story out there as well because that's what they want people to know, that you can talk. Someone is here that's willing to listen to you. It's like a family’. Hannah spoke about receiving support from patients and clinicians, not only with health concerns but with ‘what's going on with their life’, including issues such as conflict with neighbours or socioeconomic stressors that individual medical care often struggles to address.

Rose attended a yoga group regularly, in which participants were middle‐aged and elderly Black women with chronic conditions. They met weekly for medical care (provided by a white physician), yoga and peer support. In an interview, Rose explained that she was referred to the GMV by her primary care provider because ‘I'm in pain all my life’. She said she had few friends, that ‘I'm walking this earth by myself’. Rose then described the changes she experienced through attending GMVs:

I was in bad shape when I first went … It was hard, but I stuck with it, because the more I did it, the better I felt …. I like going to yoga. They make me feel welcome … All [the staff] make you feel special when you go in. … I fit in with all of them at the yoga [group]. I'm here for my health.

Despite Rose's social isolation outside of the clinic, she was now part of a health‐focused social network, made up of women who received healthcare together, practiced yoga and shared experiences of living with trauma, depression and other challenges. These peers knew when and where Rose had gone on vacation; some had shared phone numbers, rides and gym memberships. Rose's relationships with other group members had developed into what, refers to as ‘compartmental intimates’; close relationships with peers that have developed in a specific context and focus primarily on one domain, in this case, health. This development of social capital is facilitated by the frequent, consistent encounters of GMVs. Normally, trust in healthcare concerns the patient–clinician relationship. Here, the nature of GMVs allows patients to develop meaningful and trustworthy relationships with one another, too.

### 4.3 Cultivating Clinician Cultural Health Capital in Group Medical Visits

Another novel effect of group medical visits on capital formation is the potential for clinicians to foster, then mobilise their own cultural health capital. GMV clinicians are participants in the health‐focused social network, sometimes taking information and resources shared by GMV patients and using them in individual encounters, thereby mobilising social capital from a network primarily made up of patients and converting social capital into clinician CHC. A staff member summarised the benefits of GMVs for clinicians as, ‘more time, more exposure, people are hearing things they wouldn't hear in the exam room’. By virtue of listening to patients, clinicians learn things that they may not have thought to ask about. They can use this knowledge in future interactions in ways that enhance their relationships with their patients and may improve care. In this way, clinicians leverage social capital afforded to them through the GMV in similar ways as their patients. Staff members spoke briefly about their own health goals and received support from colleagues and patients in ways that many patients spoke positively about. One patient said that he found it ‘cool’ when the staff spoke about their own lives. ‘That means they're not judging anybody; they're participating also, which makes the group stronger’. The knowledge that clinicians acquire from patients in GMVs can have compound effects as they put it to use with other patients in individual or group settings. The potential for that knowledge to be taken up by future patients means that clinician CHC is also cultivating those patients' CHC. One type of new knowledge clinicians acquired in GMVs relates to the context of health‐related behaviours. The group setting and extended time shaped how they received this information, as with Zoya's disclosures about medication use. A key place where this happened was when patients spontaneously shared information that may not initially appear to be clinically relevant, but ultimately impacts their health.

GMVs also promoted shifts in clinician–patient power dynamics. In one diabetes GMV, many patients used insulin, which can only be taken by daily injections. One patient new to using insulin cried while sharing with the group that she was so afraid to inject herself that she had a neighbour come sit with her several times. Another patient who was a long‐time insulin user told the group that she hated giving herself injections. She shared a story about calling a mental health crisis hotline late one night because she needed support convincing herself to inject her insulin. While these patients spoke and others sympathised, Mary, an experienced GMV clinician, listened quietly. At the end of the conversation, she said that despite many years of clinical practice, she had never thought about how hard it could be to use insulin. In a later conversation, Mary reflected on how that day completely changed the way she talked with patients about insulin. Instead of assuming it was a simple process akin to starting other new medications, she viewed it as a substantial transition that would require daily commitment and could cause intense emotions and need for support. Mary used this information—surfaced by GMV patients, shared and corroborated among them—in subsequent interactions with *other* patients to be more understanding, proactively anticipate their fears, and troubleshoot challenges with daily injections. In a later GMV session, Mary told the group members how the insulin conversation had affected her.

We argue that Mary's new understanding of insulin use became part of her CHC, with potential to benefit her future patients initiating insulin therapy. This information only came to light because of the structure of the GMV. One patient felt comfortable disclosing her experience, creating space and opportunities for others to share similar experiences. The shift in power relations made possible by the GMV structure narrowed the gap between Mary's position of power as a clinician and the positions of her patients.

Mary's learning was cemented by the presence and concordance of multiple patients, confirming the significance and relevance of new knowledge. What Mary learnt from her patients about the context of their health behaviours (in this case, challenging experiences with medication use) became part of Mary's CHC, with the potential over time to shift her habitus. Specifically, it translated into different and arguably more patient‐centred practices that she used in future healthcare interactions (both individual and group visits). These practices and Mary's fuller understanding in turn could likely strengthen her relationships with her patients and perhaps improve their health. This shift also advantaged Mary, who may now be seen by her patients as a more understanding and skilful clinician. If Mary's skill at supporting patients improves their health—if, for example, they use insulin consistently and their blood sugar is lower—future clinicians may also see them as ‘good patients’ due to their adherence to treatment. Because GMVs are typically longitudinal, there are opportunities for clinicians to reflect back to patients how they may have changed their practice, legitimating patients' sense of GMVs as settings in which their knowledge and experiences are valued and prioritised.

In other cases, clinicians related that what they learnt about patients thanks to the extended time and peer presence in GMVs was in fact useful to themselves *and* other clinicians treating the same patients. Rohan explained:

[GMVs] are really enriching, for me certainly, and I hope for [patients], because I know them so much better …. We have the time and safe space to go into more depth around trauma, and life history, and choices, and how that relates to food and medication adherence … I will often send a summary after a group of each patient to the primary [care provider]. I try and encapsulate what have I learnt from [the patient] …. This person's brother died of kidney disease, so he's very nervous about all medicines damaging his kidneys, which is why he doesn't take any of his pills …. [Among] my panel of 1,000 patients, 100 of them have been through my groups. It's a much richer relationship.

Rohan's patients benefitted from his increased understanding of their health experiences while they participated in the group, but also longer‐term. Group visit clinicians use knowledge learnt in GMVs in their own practice, and by transmitting this knowledge to other clinicians, mobilise social capital in ways that can help their colleagues provide better care.

## 5 Discussion

This article examines the cultivation and mobilisation of capital in group medical visits, a healthcare field that provides an empirical context to study lay–expert relations and multidirectional knowledge flows. Because GMVs involve multiple patients and staff, they constitute emergent social networks through which information shared by a single patient can be taken up and acted on by group participants, including staff. Because the group's social capital is available to all, and because collaborative problem‐solving is fundamental to GMVs, they heighten the chance for social capital to be accrued and then deployed in the GMV or individual healthcare encounters. GMV staff foster these networks of social connections, in which individuals can exchange the kinds of informational and emotional support that prior research has found beneficial for health (Umberson, Crosnoe, and Reczek 2010; Perry and Pescosolido 2015). These findings mirror those from other forms of group‐based health services that have also been shown to offer benefits, including support groups (Brunelli, Murphy, and Athanasou 2016), group therapy (Leszcz 2020) and social prescribing efforts that include peers (Stickley and Hui 2012). Research on social capital and cultural health capital has demonstrated that context matters for its cultivation and deployment (Shim 2010; Chang, Dubbin, and Shim 2016; Rubin et al. 2018). Here, we demonstrate that the structure of the GMV field (e.g., grouping peers together, longitudinal relationships and extended time) can assist clinicians in cultivating patient CHC. Our findings suggest that GMVs may lessen the dependence on individual clinicians' and patients' CHC while providing opportunities for clinician and patient CHC to be cultivated in ways that are made possible in large part by the GMV format and structure. Additionally, when clinicians invest in learning how to be GMV facilitators, it also benefits the individual care they provide. In long‐term GMVs, there may even be the development of a collective habitus among all participants, patients and clinicians alike, who come to know each other well and share experiences over time.

It is worth noting that we observe this in a setting serving people with limited economic resources. GMVs represent opportunities to form and build organisationally embedded ties. As argues, these sorts of ties permit ‘the cumulative effects of small benefits—a free service in one setting, a crucial bit of information in another, a valuable discount in a third, a comforting ear in a fourth—that accrue’. In settings where healthcare resources are limited, yet disproportionately provide care to those who have less access to multiple sorts of capital, extended time and group support may be especially beneficial for increasing social and cultural health capital. Cultural health capital aims to understand how healthcare inequalities are reproduced through clinical interactions, with implications for understanding how racism and other forms of discrimination occur within daily healthcare practice (Logan et al. 2021). However, our findings on the effects of grouping together patients with similar medical conditions and shared social experiences, along with the structuring of sessions to lower the risks of disclosure, shows how the consequences of unequal CHC might be mitigated in GMVs.

In addition, we observe spillover effects, from patients to others in their networks, from GMV facilitators to other healthcare professionals and from one group at one point in time to other group or individual clinical interactions. Group visits therefore provide organisational settings in which both bonding social capital (among participants) and bridging social capital (across typical patient–clinician boundaries and to other networks) can be forged and mobilised (Pitkin Derose and Varda 2009). In typical healthcare encounters, knowledge and information is seen as being disseminated by expert clinicians to patients, unidirectionally. In contrast, GMV patients share information and suggestions that staff can learn from, potentially adding approaches to communication or local resources to their clinical repertoire. For Zoya and other patients, time with trustworthy clinicians, community health workers and peers led to consistent medication use and learning about chronic disease management in ways likely to benefit their health long‐term. For Mary and other clinicians, cultivation of their own CHC is possible because there is time for problems and information to surface as clinically relevant. For example, conversation about health practices such as insulin use deepens clinician understanding of challenges that may disrupt follow‐through with treatment recommendations. Thus, clinicians experience opportunities to accumulate their own CHC—beneficial for both GMVs and individual care—by benefitting from a patient‐focused social network while simultaneously working to cultivate patients' CHC.

The role of peers in GMVs means that clinician habitus may play less of a role in determining what kind of care is offered. When clinicians are outnumbered by patients, they do not unilaterally dictate the rules of the game. For example, Monica McLemore has proposed that the GMV format may increase patient safety because clinicians may be less likely to discriminate against members of marginalised groups when they are being observed by multiple other patients (DiGregorio 2020). Additionally, the extended time in GMVs allows for multiple kinds of interactions including peers advocating for each other (Thompson‐Lastad 2018). Clinicians working in the GMV field adjust to its rules and accrue particular forms of CHC that allows them to successfully work in the field. Over time, clinicians who continue working in GMVs might experience shifts in habitus that align well with this field. Additionally, racially concordant GMVs are increasingly being implemented, particularly in group prenatal care, and could be an additional empirical site for deepening understanding of the effects of CHC and racism on healthcare.

It is important to note the central impact of the norms of the GMV field—the high ratio of patients to clinicians, encouragement of patients to share their experiences, expectations that facilitators ‘step back’, and visit frequency and extended time—as well as the accompanying organisational resources needed to offer GMVs. Though providing a few GMVs per week is feasible in many settings, moving a substantial amount of care into GMVs would require additional infrastructure, including staff training and rearranged clinic space. Making these organisational changes could be part of broader efforts to address medical racism and other barriers to equitable healthcare.

Such organisational and systemic interventions can also fundamentally impact another major issue in the social organisation of contemporary healthcare: the growing problem of clinician burnout, caused in part by working conditions (National Academies of Sciences, Engineering, and Medicine 2019; Panagioti et al. 2017). Negative working conditions known to contribute to burnout include brief visits and what many perceive to be the conveyor belt nature of conventional patient care (Satterwhite 2019; Thompson‐Lastad and Gardiner 2020). Several clinicians in this study felt so negatively affected by the limited time in individual visits that they had stopped doing individual primary care. Two convinced clinic administrators to allow them to exclusively practice in GMVs because they saw it as their best means of avoiding burnout. Conversely, other interviewees have since left safety‐net primary care.

The potential benefits of GMVs for the cultivation and deployment of social and cultural health capital surely vary by clinician identity, patient population, care setting, and more. In this respect, this study does have several limitations. First, we did not collect data about individual care at the clinics in this study, which would have allowed us to directly compare individual and group care. Second, though the study includes representation from 20 distinct GMVs in two languages across four organisations, participants do not represent the full array of patients and staff participating in group visits. Longitudinal ethnographic data following GMV patients and staff in multiple care settings would best illuminate how social capital and CHC are deployed and leveraged in subsequent healthcare interactions. Finally, all sites in this study were in US states that had expanded Medicaid, the public insurance programme for those with limited income, a policy shift that substantially increased access to primary care. Dynamics observed might be distinct in primary care settings that primarily serve privately insured patients, or settings where more patients are uninsured and have especially limited access to care.

This study of GMVs shows that organisationally embedded settings can change the interpersonal dynamics of patient–clinician relationships and disrupt hierarchical flows of information. This can then produce important shifts in the social and cultural capital available to those who participate, as well as improvements in clinician work satisfaction. Group visits' combination of medical care, social connection and access to capital is particularly critical for those experiencing social disadvantage or isolation. They constitute a rare acknowledgement that the investment of time and resources to accumulate the capital necessary to successfully navigate the healthcare system is highly stratified. Patients are clearly aware of this: One participant, Luis, had infrequently accessed care for his diabetes until he was invited to a monthly GMV for Spanish‐speaking adults under 50 living with diabetes. The group met in the evenings and included dinner, medical care with a bilingual doctor and discussion with peers (often with family members in attendance). When the staff announced that current members would return to individual care so that new patients could participate, Luis was clear: he would not be coming to the clinic for individual care. Why, he reasoned, would he take off work to wait in the waiting room for an hour and spend only 10 min with a doctor? In Luis' view, because accessing healthcare is exceedingly complex and presents obstacles and administrative burdens (Herd and Moynihan 2019) that are exhausting to overcome, healthcare institutions bear significant responsibility for generating and even magnifying health inequities. Group medical visits offer one opportunity for healthcare institutions to mitigate the inequalities they otherwise produce.^2^

## Author Contributions

**Ariana Thompson‐Lastad**: funding acquisition, conceptualisation, methodology, investigation, writing–original draft. **Jessica M. Harrison**: validation, writing–review & editing. **Janet K. Shim**: formal analysis, writing–review & editing.

## Ethics Statement

This study was approved by the University of California, San Francisco's Human Research Protection Programme Internal Review Board (IRB # 15‐18421).

## Consent

Research participants were fully informed, verbally and with written materials, of the study's purpose and procedures. All participants consented to participate.

## Conflicts of Interest

The authors declare no conflicts of interest.

## Permission to Reproduce Material From Other Sources

N/A.

## Data Availability

The data that support the findings of this study are available on request from the corresponding author. The data are not publicly available due to privacy or ethical restrictions.

## Endnotes

^1^
We use the term ‘clinician’ to refer to licenced healthcare providers, and ‘staff’ to refer more broadly to all healthcare workers.

^2^
The clinic staff relented and allowed Luis and his peers continue attending the monthly GMVs—until a change of clinic administration ended the diabetes group visit programme entirely.
